# Síndrome de Ramsay-Hunt: a propósito de dos casos en que se identificó el genoma del virus de la varicela-zóster en líquido cefalorraquídeo

**DOI:** 10.7705/biomedica.5985

**Published:** 2021-12-15

**Authors:** Marcelo Corti, María F. Villafañe, Jorge Correa

**Affiliations:** 1 Departamento de Medicina, Orientación Enfermedades Infecciosas, Facultad de Medicina, Universidad de Buenos Aires, Buenos Aires, Argentina Universidad de Buenos Aires Departamento de Medicina, Orientación Enfermedades Infecciosas Universidad de Buenos Aires Buenos Aires Argentina; 2 División VIH-Sida, Hospital de Infecciosas “Francisco Javier Muñiz”, Buenos Aires, Argentina Hospital de Infecciosas “Francisco Javier Muñiz” Buenos Aires Argentina

**Keywords:** herpes zóster, HIV, síndrome de inmunodeficiencia adquirida, parálisis facial, líquido cefalorraquídeo, Herpes zoster, HIV, acquired immunodeficiency syndrome, facial paralysis, cerebrospinal fluid

## Abstract

Como los otros herpesvirus alfa, el virus de la varicela-zóster (VZV) permanece en estado de latencia en los ganglios neurales después de la infección primaria (varicela). La reactivación de una infección latente por VZV en los ganglios de la raíz dorsal, produce el herpes zóster. La erupción que este provoca se caracteriza por lesiones cutáneas metaméricas que se acompañan de dolor neurítico y comprometen con mayor frecuencia a ancianos y sujetos inmunocomprometidos, en especial, aquellos infectados con el virus de la inmunodeficiencia humana (HIV).

Las complicaciones que se observan en esta población de pacientes incluyen neumonía, hepatitis y compromiso del sistema nervioso central (meningitis y encefalitis). Varios síndromes clínicos se asocian con el herpes zóster de localización craneal, incluida la parálisis facial periférica y el síndrome de Ramsay-Hunt, el cual constituye la segunda causa de parálisis facial periférica y tiene una gran variedad de presentaciones clínicas. La parálisis facial se presenta en 60 a 90 % de los casos de síndrome de Ramsay-Hunt, puede preceder o aparecer después de las lesiones cutáneas y tiene peor pronóstico que la parálisis de Bell.

Se describen aquí dos casos de herpes zóster del ganglio geniculado, con parálisis facial periférica que coincidió con la aparición de las lesiones cutáneas vesiculosas en el conducto auditivo externo y el pabellón auricular (síndrome de Ramsay-Hunt multimetamérico). En ambos casos, se identificó el genoma del VZV mediante PCR en el líquido cefalorraquídeo (LCR).

## Abstract (en)

Like other alpha-herpesviruses, the varicella-zoster virus (VZV) remains latent in the neural ganglia following the primary varicella infection. The reactivation of the VZV in the dorsal root ganglia results in herpes zoster. Herpes zoster eruption is characterized by localized cutaneous lesions and neuralgic pain mostly in older and immunocompromised persons, especially those living with the human immunodeficiency virus (HIV).

The most commonly reported complications include VZV pneumonia, meningitis, encephalitis, and hepatitis. Several neurologic syndromes have been described associated with herpes zoster localized in cranial areas including peripheral nerve palsies and the Ramsay-Hunt syndrome, which has a varied clinical presentation and is the second most common cause of peripheral facial paralysis. Facial paralysis in this syndrome occurs in 60 to 90% of cases and it may precede or appear after the cutaneous lesions with a worse prognosis than idiopathic Bell paralysis.

Here we present two cases of herpes zoster from the geniculate ganglia with peripheral facial paralysis that appeared simultaneously with vesicular herpetic otic lesions (multimetameric Ramsay-Hunt syndrome). In the two cases, amplifiable varicella-zoster viral DNA was found in the cerebrospinal fluid by RT-PCR Multiplex.

El virus de la varicela-zóster (VZV) o virus del herpes humano de tipo 3 (HHV-3) pertenece al género *Varicellovirus*. El genoma es el más pequeño de todos los miembros de la familia Herpesviridae. Las especies de esta familia se agrupan en tres subfamilias según las características de su genoma, estructura, tropismo tisular, efecto citopático y las células en las que se mantienen en estado latente. Dichas subfamilias se denominan Herpesvirinae alfa, beta y gamma. El VZV pertenece a la Alfaherpesvirinae ^1^.

Cuando el VZV ingresa por primera vez al organismo de un huésped sensible, produce la varicela de patogenia exógena, que se caracteriza por una erupción máculo-papular y vesicular costrosa, cuya primera modalidad de diseminación es la hemática (viremia). La segunda forma clínica, o reactivación endógena, es el herpes zóster, que ocurre luego de un período variable de latencia en las células neurales (neuronales y gliales) de los ganglios sensoriales. Esta afección se observa predominantemente en pacientes inmunocomprometidos y en ancianos cuando el virus migra por vía axonal hasta la piel, provocando una erupción, generalmente metamérica y, con frecuencia, diseminación a otros órganos como pulmón o el hígado y el sistema nervioso central ^2^.

## Casos clínicos

### Caso 1

Se trata de un varón de 46 años con antecedentes de HIV/sida en tratamiento de mantenimiento, y criptococosis y tuberculosis meníngeas en tratamiento de consolidación. Ingresó al Hospital de Infecciosas “Francisco Javier Muñiz” (Buenos Aires) por presentar lesiones máculopapulares vesiculosas localizadas en la hemicara derecha, el pabellón auricular y el dorso de la lengua homolaterales indicativas de herpes zóster multimetamérico. El paciente había iniciado recientemente el tratamiento antirretroviral de gran actividad (TARGA) con un esquema de 50 mg/día de dolutegravir, 200 mg/día de emtricitabina y 300 mg/día de tenofovir desoxifumarato. El recuento de linfocitos T CD4+ fue de 134 células/μl (8 %).

A su ingreso, se le practicó una tomografía computarizada (TC) de cerebro y se descartaron lesiones ocupantes de espacio; además, se le practicó una punción lumbar. Se obtuvo líquido cefalorraquídeo (LCR) claro, y se detectó una discreta pleocitosis (8 células/ml, 100 % mononucleares), glucorraquia de 40 mg/dl, e hiperproteinorraquia de 1,88 g/L. La reacción en cadena de la polimerasa para herpes virus en líquido cefalorraquídeo (PCR multiplex) resultó positiva para el genoma de VZV y negativa para el resto de los herpesvirus.

Se diagnosticó síndrome de Ramsay-Hunt en estadio I y se inició el tratamiento con aciclovir intravenoso en dosis de 2 gramos por día durante 14 días. Se obtuvo una buena evolución clínica y mejoría de las lesiones de piel y mucosas. Se descartó compromiso ocular y auditivo y, dado que el paciente evolucionó favorablemente, se le dio de alta para su seguimiento por consulta ambulatoria.

### Caso 2

Se trata de un varón de 55 años con diagnóstico de HIV/sida de larga data, con deficiente cumplimiento del tratamiento antirretroviral de gran actividad (TARGA), el cual había reiniciado dos meses antes con un esquema de 50 mg/día de dolutegravir, 200 mg/día de emtricitabina y 300 mg/día de tenofovir desoxifumarato.

**Figura 1:**
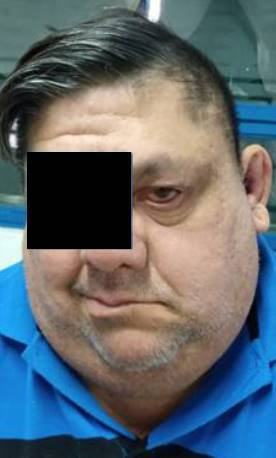
Parálisis facial periférica izquierda con imposibilidad para el cierre palpebral y caída de la comisura labial

**Figura 2:**
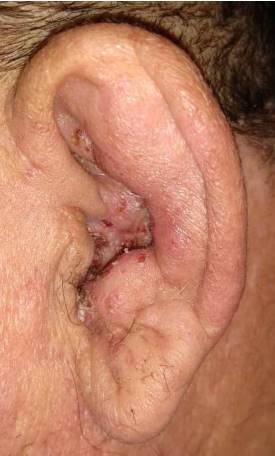
Erupción vesicular costrosa por herpes zóster en el conducto auditivo externo y el pabellón auricular

Consultó por otalgia, *tinnitus*, paresia moderada en hemicara izquierda y dificultad para cerrar el ojo (figura 1), además de lesiones vesiculosas costrosas en el pabellón auricular homolateral de 15 días de evolución (figura 2). El cuadro clínico se interpretó como una erupción por VZV con compromiso de más de un par craneal y síndrome de Ramsay-Hunt en estadio III.

En el examen físico, se constató la presencia de paresia moderada en la hemicara izquierda, con dificultad para cerrar el ojo, y lesiones vesiculosas costrosas en el pabellón auricular homolateral.

El recuento de linfocitos T CD4+ fue de 111 células/μl (3 %) y la carga viral para HIV era indetectable: <40 copias/ml (log_10_<1,60). Se practicó una TC de cerebro con la cual se descartaron lesiones ocupantes de espacio. Se efectuó una punción lumbar y se obtuvo líquido cefalorraquídeo claro, normotenso, con 5 células/ml, glucorraquia de 74 mg/dl y proteínas de 0,33 g/L. La PCR multiplex en el líquido cefalorraquídeo resultó positiva para el genoma del VZV y negativa para el resto de los herpesvirus.

Se inició el tratamiento con aciclovir intravenoso en dosis de 2 g por día durante 14 días, con buena evolución clínica y mejoría de las lesiones de piel y mucosas. Durante la hospitalización, el paciente presentó falla renal y síndrome vertiginoso, por lo que se agregaron corticoides en dosis diarias de 1 mg/kg, y requirió cuidados intensivos para el manejo de la función renal durante 72 horas. Se completó el tratamiento con aciclovir intravenoso durante 14 días y, al finalizarlo, se hizo una nueva punción lumbar de control obteniéndose 3 células/ml, glucorraquia de 102 mg/dl, y proteínas de 0,35 g/L. La PCR para VZV resultó negativa. Dada la mejoría clínica y de la función renal, se dio de alta al paciente para continuar con el seguimiento y el control ambulatorios.

### Consideraciones éticas

Se obtuvo la autorización de ambos pacientes para publicar los casos clínicos, y la del paciente del caso 2 para la publicación de las fotos e imágenes.

## Discusión

El IX par craneal, o nervio glosofaríngeo, se origina en el ganglio geniculado, en la superficie lateral del bulbo, cerca del primer codo del VII par craneal y de los núcleos de origen de los nervios vago (X par) y espinal o accesorio. Contiene fibras motoras y sensoriales: las motoras inervan el músculo estilo-faríngeo y el músculo constrictor superior de la faringe, y las sensoriales transmiten la sensibilidad general de la orofaringe y la sensación gustativa del tercio posterior de la lengua.

El herpes zóster ótico, descrito por Ramsay y Hunt en 1907, es un síndrome que conjuga la parálisis facial periférica y la erupción cutánea pápulo-vesiculosa, la cual puede comprometer la membrana timpánica y el conducto auditivo externo homolateral. En ocasiones, la erupción se extiende al pabellón auricular ipsilateral o la mucosa oral como consecuencia de la afectación del ganglio geniculado por el VZV ^3^. La parálisis facial es muy parecida a la llamada parálisis de Bell. La afectación de los ganglios de Corti y Scarpa se acompaña de vértigo, *tinnitus*, nistagmo, náuseas, vómitos e hipoacusia neurosensorial debido al compromiso del VIII par craneal por proximidad ^4^. Se estima que este síndrome incluye entre el 7 y el 16 % del total de las parálisis faciales periféricas unilaterales no traumáticas; la afectación facial suele aparecer entre los días 4 y 15 después de la erupción, y su evolución es más grave que la de la parálisis facial de Bell. La recuperación completa solo se alcanza en el 50 % de los adultos ^5^.

Los sujetos inmunodeprimidos son especialmente proclives a desarrollar esta enfermedad, sobre todo aquellos que reciben tratamiento inmunosupresor, individuos infectados por el HIV (como los casos que se describen) y los portadores de enfermedades hematológicas malignas ^6^. La presentación clínica es muy variada. La enfermedad se clasifica en cuatro estadios, del I al IV. El estadio I incluye otalgia y erupción de vesículas en el territorio del nervio facial; el estadio II incluye, además, parálisis facial periférica homolateral; el estadio III o síndrome de Sicard comprende la triada de dolor, erupción y parálisis facial, así como acúfenos e hipoacusia perceptiva de difícil recuperación y, a veces, episodios de vértigo; el estadio IV se caracteriza por la afectación de otros pares craneales, en especial el V par, por lo que se trataría de una polineuropatía craneal ^7^. La presentación del síndrome de Ramsay-Hunt como una polineuropatía de varios pares craneales fue descrita por Aviel y Marshak ^7^, quienes comprobaron la participación de los siguientes nervios en orden decreciente: VII, VIII, IX, V, X y VI, siendo rara la participación del resto de los pares craneales.

Las manifestaciones clínicas dependen del territorio o territorios que inervan los pares craneales afectados. Su característica esencial es la de ser estrictamente unilateral, deteniéndose en la línea media, como toda erupción por herpes zóster. La sobreinfección de las lesiones, sobre todo las cutáneas, es frecuente. En la mayoría de los casos el diagnóstico es clínico y se basa en la presencia de las lesiones cutáneas típicas en el oído externo y, a veces, con carácter metamérico, en otras zonas de la cabeza y el cuello ^6^. En general, la erupción vesicular precede a la parálisis facial, pero en el 14 % de los casos esta es posterior y complica el diagnóstico ^8^ En otros pacientes, las vesículas herpéticas no llegan a aparecer, forma clínica que se conoce como *zoster sine herpete* y que, según algunos estudios, corresponde al 16 % del total de las parálisis faciales periféricas ^9^. Una pequeña proporción de los pacientes con un diagnóstico inicial de parálisis idiopática de Bell, podrían sufrir en realidad un *zoster sine* herpete o síndrome de Ramsay-Hunt atípico ^6^^,^^10^.

Aunque el diagnóstico es eminentemente clínico, es posible detectar serologías específicas para el VZV hasta en el 25 % del total de parálisis faciales. Mediante PCR, se ha podido detectar el genoma del VZV en el 89 % de los casos estudiados de síndrome de Ramsay-Hunt, pero son técnicas que confirman el diagnóstico tardíamente y no están disponibles en todos los centros médicos ^6^. La única prueba útil para evaluar las lesiones cutáneas, por su rapidez de ejecución y sencillez de interpretación, es el citodiagnóstico de Tzanck ^11^.

Cualquier persona que haya padecido varicela puede presentar un síndrome de Ramsay-Hunt. La reactivación del VZV puede causar varicela en los contactos si estos no la han tenido o no han sido vacunados previamente.

Como tratamiento, los antivirales más aconsejables son: aciclovir, 30 mg/ kg/día de 10 a 14 días; famciclovir, 500 mg cada 8 horas durante 7 días, o valaciclovir, 1 g cada 8 horas durante 5 días. Estos dos últimos tienen la ventaja de que el tratamiento es de una menor duración y con dosis menos frecuentes ^12^.

En cuanto al uso de corticosteroides en estos casos, en el estudio retrospectivo de Coulson, et al. ^13^, sobre los factores de pronóstico en 101 pacientes con síndrome de Ramsay-Hunt, los autores concluyeron que su uso temprano conjuntamente con el tratamiento antiviral tuvo mayores beneficios en comparación con no emplearlos. Una explicación posible de este hallazgo es que el efecto antiinflamatorio de los esteroides es más beneficioso en el momento de mayor inflamación de los nervios craneales por la acción del VZV. En los dos casos que se describen, el uso de esteroides fue necesario, especialmente en el paciente número 2 por el gran componente vertiginoso que formaba parte del cuadro clínico. En ambos se utilizó la formulación intravenosa de aciclovir al detectar el genoma viral en líquido cefalorraquídeo mediante PCR y la evolución fue muy favorable.

## Conclusión

En conclusión, las complicaciones neurológicas del VZV son mucho más frecuentes en los pacientes inmunocomprometidos y, especialmente, en los infectados con el HIV. En estos casos, se observan formas cutáneas diseminadas que simulan una varicela y compromiso visceral, incluido el sistema nervioso central. En este sistema, lo más común es el compromiso vascular (vasculitis/angeítis granulomatosa de grandes vasos), que puede simular un accidente cerebrovascular agudo (encefalitis de grandes vasos), y la hemiparesia contralateral tardía, denominada así porque ocurre generalmente algunas semanas después de un episodio de herpes zóster con afección de la primera rama del V par (trigémino) ^14^^-^^16^. El compromiso de las meninges, comprobada en los dos casos que se presentan, es muy infrecuente y está escasamente citada en la literatura médica ^17^^,^^18^.
